# Dementia awareness among elderly at risk for developing mild cognitive impairment: a cross sectional study at a university-based primary care clinic

**DOI:** 10.1186/s12877-023-04230-4

**Published:** 2023-08-17

**Authors:** Mohd Fairuz Ali, Nur Iman Suraiya Ja’afar, Thayaletchumy Gophala Krishnan, Mohamad Azizi Mohamad Zulkifle, Nur Khairunnisa Khaidzir, Teh Rohila Jamil, Zuraidah Che Man, Aznida Firzah Abdul Aziz

**Affiliations:** 1 Department of Family Medicine, Faculty of Medicine, Universiti Kebangsaan Malaysia, Jalan Yaacob Latif, Bandar Tun Razak, Cheras, Kuala Lumpur, 56000 Malaysia; 2 Class of 2021/2022, Department of Family Medicine, Faculty of Medicine, Universiti Kebangsaan Malaysia, Jalan Yaacob Latif, Bandar Tun Razak, Cheras, Kuala Lumpur, 56000 Malaysia; 3 Quality Service Unit, Department of Emergency Medicine, Faculty of Medicine, Universiti Kebangsaan Malaysia, Jalan Yaacob Latif, Bandar Tun Razak, Cheras, Kuala Lumpur, 56000 Malaysia

**Keywords:** Dementia, Cognitive impairment, Aged, Awareness

## Abstract

**Background:**

The number of people living with dementia in Malaysia is expected to increase with the nation’s growing elderly population and increased lifespan. The lack of public awareness of dementia is partly compounded by low personal health literacy, while scarce research on local patient awareness further impacts the execution of optimised healthcare services in Malaysia. Patients with chronic disease have an elevated risk of developing mild cognitive impairment (MCI). This study aimed to assess the level of awareness of basic knowledge on dementia among the elderly, especially those at risk of developing mild cognitive impairment and its associated factors.

**Methods:**

A total of 207 elderly patients aged 60 years and above with chronic diseases attending a university-based primary care clinic were recruited via a systematic randomised sampling method from the clinic patient attendance registry. Respondents were assessed using self-administered online questionnaires distributed via mobile devices. The questionnaire assessed awareness, i.e. ability to correctly answer a self-reported questionnaire on basic dementia knowledge; (adapted from Northern Ireland Life and Times Survey 2010), risk of MCI; (using Towards Useful Aging (TUA)-WELLNESS screening questionnaire) and help-seeking behaviour. Bivariate analysis was used to determine factors associated with dementia awareness.

**Results:**

The response rate was 77.1%, with the majority of participants were females, Chinese and had secondary school education. 39.1% of participants were categorised as high risk of developing MCI. The majority (92.8%) had low dementia awareness and had never shared their concerns regarding dementia (93.2%) nor had any discussion (87.0%) on cognitive impairment with their physicians. Three factors had an association with total dementia awareness score, i.e., younger age group, higher risk of MCI and presence of cardiovascular diseases have significantly lower awareness score (*p* < 0.05).

**Conclusion:**

Awareness of dementia is low among elderly patients with potentially high risk of developing MCI. Efforts to improve awareness on dementia should focus on primary care doctors engaging with at-risk elderly patients to initiate discussion regarding dementia risk while managing modifiable risk factors i.e. hypertension control, diabetes, dyslipidaemia and obesity.

## Background

The term “population aging” refers to changes in the age composition, such as an increase in the proportion of elderly people [1]. It is predicted around 75 million individuals worldwide will have dementia in 2030, with the majority residing in low and middle-income nations [2]. The number of individuals living with dementia worldwide is projected to triple from 50 million in 2018 to 152 million in 2050 as the world’s population ages [3].

Malaysia is fast progressing to become an aging population, given the current trends in life expectancy and birth rate [4]. The proportion of the elderly is projected to reach 16.3% of the total population by the year 2040 [5]. It is estimated that a considerable portion of the population will be in danger of cognitive decline and dementia later in life. By 2050, the approximate total number of people living with dementia (PLWD) in Malaysia will rise to 590 000 people [6]. However, statistically predicted trends may not reflect the actual burden of PLWD in this country.

The possible complications of dementia include loss of ability to function or care for self and inability to communicate with others. As the disease progresses, PLWD will experience difficulty solving problems and forgetting recent events. As there is no curative treatment for dementia, prevention and the proactive management of modifiable risk factors to delay or slow the onset or progression of the disease are key action areas in the WHO Global Action Dementia Plan [2]. For these reasons, controlling these modifiable risk factors is essential and best addressed in primary care. Findings from studies on dementia risk prediction models provide an effective and simple way of identifying individuals who would benefit from intervention to reduce their dementia risk. Earlier research have focused on the accuracy of predictive models for dementia in high-income countries (HIC) and is now tested for its applicability in low-income countries (LIC) [7]. For LIC, predictive modelling suggests that dementia shares the same risk factors as cardiovascular disease, which are known causes of stroke and subsequent sequelae of vascular dementia. There are twelve potentially modifiable risk factors for dementia which include less education, hearing loss, traumatic brain injury, alcohol consumption, obesity (body mass index ≥ 30), hypertension, smoking, depression, social isolation, physical inactivity, air pollution and diabetes [7].

A study in Northern Ireland showed only a low percentage (29%) of people were aware of the preventive measures for dementia and the link between awareness of dementia and healthy lifestyles, such as diet and exercise [8]. This is usually addressed in the primary care setting. Consultation on risk factors and diagnosis of cognitive problems should be initiated at the primary care level. According to the Lancet, around 35% of dementia is caused by a combination of nine modifiable risk factors, i.e., low education level, midlife hypertension, obesity, hearing loss, as well as preventing diabetes, smoking, depression, physical inactivity, and social isolation at a later life [9]. Certain risk factors may substantially impact dementia development at a specific point in a person’s life and should be addressed at those particular times [9].

Preventive measures for dementia should begin once patients demonstrate early symptoms or signs of the disease, especially among those with potentially high risk. Mild cognitive impairment (MCI) precedes the onset of dementia. The likelihood of progression from MCI to any form of dementia has been suggested to occur at a rate 3 to 5 times higher than those with normal cognition, with an annual rate of progression of 12% in the general population and up to 20% in people at higher risk [10]. Based on a study conducted among participants enrolled in the prospective Mayo Clinic Study of Aging, over a median follow-up of 5.1 years, 28.7% of participants with prevalent or incident MCI progressed to dementia (71.3 per 1,000 person-years) [11]. The typical MCI characterisation mentioned by Petersen includes the following characteristics: cognitive decline not normal for age, memory disorder, ability to perform daily activities, intact general cognitive skills, objective evidence of a memory deficit, and absence of dementia [12]. However, the criteria of diagnosing MCI has evolved throughout the years beyond memory impairment and involved different aetiologies and pathophysiologic biomarkers [13]. Evidence suggests that people with MCI are at a higher risk of dementia, especially if they have associated medical disorders [14]. The early discovery of dementia risk factor(s) will enable secondary prevention through optimised risk factor control [10].

Dementia awareness is an essential factor in dealing with dementia. It could be a factor that drives individuals with Dementia (PLWD) to seek medical treatment and intervention [15]. The misconception that dementia is a normal aspect of aging rather than a condition that must be treated is a key reason for the lack of awareness of dementia [16]. Another common misconception is the lack of knowledge on risk factors for developing dementia and the role of prevention [15]. Apart from the patients and their caregivers, the awareness of dementia is equally essential for healthcare providers to ensure timely secondary prevention measures.

Dementia is globally underdiagnosed or is often diagnosed at a relatively late stage of the disease process [2]. A study on dementia detection rates in selected public primary care clinics in Malaysia observed that the failure of primary healthcare providers to identify patients with dementia may be due to poor knowledge leading to diagnosing dementia [17]. Based on a report from the Royal Australian College of General Practitioners, only 6% of GPs believe dementia is a growing health concern, implying that GP awareness and knowledge of dementia should be improved [18]. Another factor could be the existing negative stigma of dementia, especially in the conservative Asian society. Anecdotal reports to Dementia Australia suggest that dementia is frequently dismissed as a normal part of aging even by medical health professionals [18].

Lack of awareness and knowledge can lead to dementia-related stigma, labelling, stereotyping, separation, loss of social status, discrimination towards PLWD and people supporting someone with dementia [19]. Dementia-related stigma can cause significant adverse effects such as low self-esteem, isolation, poor mental health, and decreased quality of life in PLWD. It has also been identified as one of the most critical factors contributing to the avoidance of help-seeking behaviours, delaying the diagnosis and the utilisation of health and social services [19].

Therefore, the initial steps in addressing the problem of dementia are to create awareness and improve the general public knowledge on dementia detection and management. Few earlier studies cited poor awareness of dementia within Malaysian communities [16]. However, no specific research has been done to assess the awareness of dementia among patients at risk. This study aimed to assess the level of awareness of basic knowledge on dementia among the elderly, especially those at risk of developing mild cognitive impairment and its associated factors.

## Methods

### Study design

This cross-sectional study was conducted among elderly patients attending a university-based primary care clinic (Klinik Primer PPUKM Cheras, KPPC), which provides specialised primary care services to the surrounding communities within a 10-km radius, as well as supportive shared care initiatives with the main university hospital.

Elderly patients aged 60 years old and above under follow-up for chronic care, i.e., hypertension, diabetes mellitus, hyperlipidaemia, and gout management, were recruited. Elderly patients attending the outpatient clinic for other reasons and those with an established diagnosis of dementia or other cognitive impairment were excluded. The latter group was excluded on the assumption that their awareness of dementia would influence their responses in the questionnaire.

### Recruitment of participants

A simple random sampling method using a web-based application tool (stattrek.com) to select patients from the clinic’s Appointment Registry.

**Fig. 1:**
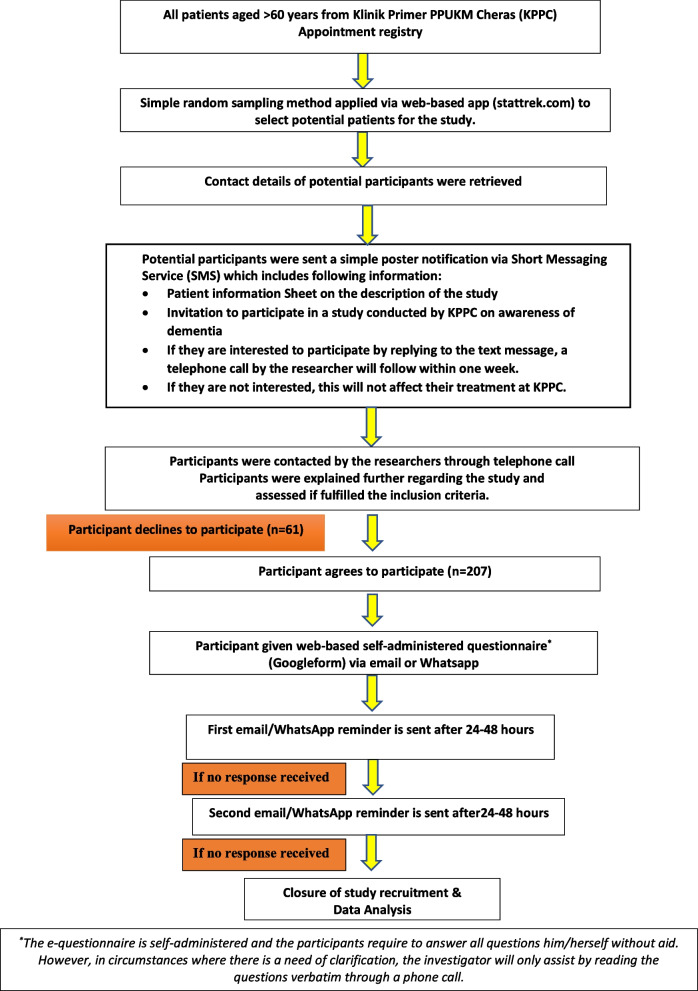
Study flow chart

Potential patients (aged 60 years old and above) were identified from the daily attendance log of the Klinik Primer PPUKM Cheras (KPPC), a university-based primary care clinic in Kuala Lumpur. This formed the sampling frame for patient selection, as there was no clinic-based elder patient registry. Simple randomised sampling was conducted using a web-based application (Stattrek.com) to select patients from the sampling frame. Potential patients were contacted via a short messaging service using a poster informing them of the study. The poster included the official telephone number of the research team to avoid rejection of an unrecognised telephone number. The patients were contacted by the researcher after 24–48 h to assess their eligibility to participate. Patients who met the inclusion criteria were invited to participate in the study. Participation is voluntary, and refusal to participate or withdrawal from the study would not affect the treatment received from the clinic. Two scheduled reminders were given via text messaging over 1 week. Participants who did not respond to both reminders were considered as declined participation. Participants who agreed to participate were required to respond to the short messaging service. Upon receiving this notification, the researcher would then proceed to contact the participants via telephone. All prospective recruits were given a patient information sheet regarding the study and their role. A written informed consent (IC) was obtained from all subjects and/or their legal guardian(s) where applicable. The IC was included together with the self-administered web-based questionnaire which was distributed via mobile devices using the *Google Form™* application. Refer Fig. 1 for details.

### Sample size

The sample size was estimated using the Leslie Kish formula, and a minimum sample of 227 participants was required, based on a precision of 0.05 and a prevalence of 16% from a study by Shahar et al. [20]. This total included an additional 10% dropout rate, which was added on to anticipate the withdrawal of participation.

### Data collection

The web-based self-administered questionnaire (Google Form™) consisted of four main sections; (i) socio-clinical demographics, (ii) awareness of dementia assessment, (iii) TUA-WELLNESS MCI risk screening tool, and (iv) consultation with healthcare providers. The questionnaire was bilingual (Malay and English) to accommodate participants with difficulty in either language. The time required to complete the questionnaire ranged from 5–10 min. In the case where assistance was required, the researcher will only assist by reading the questions verbatim through the phone.

### Outcome measures

In this study, awareness of dementia is defined as the ability to correctly answer basic knowledge statements on dementia, including a critical question, “Dementia is a part of the normal process of aging”, which the participants need to answer as false. The questions assessing dementia awareness were adapted from the Northern Ireland Life and Times (NILT) Survey 2010 [8]. The NILT 2010 is an annual survey which measures public opinion on identified themes and measures the changing public opinions since its inception in 1998 in Northern Ireland. The module on dementia was used in the 2010 survey (dementia knowledge and attitude).

Back-to-back translation of the questionnaire by Parkland et al. (2012) to the Malay language was conducted, and the contents and understandability of the questions were checked by Family Medicine Specialists who were well-versed and trained in community geriatrics and dementia care [8]. Only seven (7) out of 8 questions in Section 2 of the original NILT version covering dementia awareness were selected. During the validation process, the expert panel decided that one question on ‘drug treatments that help with dementia’ should be removed as the translated Malay version provided a similar meaning to ‘dementia can be cured’. The questionnaires consist of seven right or false statements that the participants need to answer.

The awareness of dementia was categorised into 3 levels based on the quartiles. A total score of 0 – 4, or inability to answer critical questions correctly, was categorised as low awareness; a total score of 5 indicated medium awareness, while a total score of ≥ 6 was categorised as high awareness. The total score of this section was used to look at factors associated with the awareness.

The risk of developing MCI in section (iii) of the questionnaire was assessed using the TUA (Towards Useful Aging) -WELLNESS tool developed locally and validated to address the multi-ethnic sociocultural differences in the local community [21]. It consists of 10 questions corresponding to items which cover education, fasting blood sugar (FBS) value, fasting cholesterol level, daily fruit/vegetable consumption, frequency of modern device usage, frequency of doing mechanical tasks for men, frequency of reading/sewing activity for women, frequency of practising calorie restriction, the difficulty faced in conducting daily activities, and their satisfaction of their quality of life. Each question contributes a score of 1 point, with a total score of 17. The risk of developing MCI is calculated from the total score. A score of lower than 11 indicates a high risk of developing MCI, and a score of 11 and more indicates a low risk.

### Data analysis

The data were analysed using SPSS software version 25. Descriptive analysis was presented in categorical and continuous data. Frequencies (n) and percentages (%) were used for categorical data. For continuous data, mean (x) with standard deviation (SD) and median (interquartile range) were used in nominal and non-nominal data, respectively. Bivariate statistical tests were performed using the Pearson chi-square test and Yates correction to determine the association between factors. Independent t-test analysis and Mann-Whitney U test were used for independent variables present in continuous form. *P*-value of ≤ 0.05 was set as the significance level for statistical tests.

## Results

### Background characteristics of the study participants

A total of 268 elderly patients who met the inclusion and exclusion criteria were invited to participate. A total of 207 agreed and consented to participate, making the response rate 77.24% (207/268). Among the reasons for refusing to participate were: unable to complete the questionnaire (i.e. time constraints), language barrier and unwillingness to share personal medical information despite reassurance on confidentiality.

Table 1 displays the clinical and sociodemographic characteristics of study participants. The median age of participants was 70 (IQR 9) years.

**Table 1:** Clinical and socio-demographic characteristics of study participants (*N* = 207)

**Independent variables**	**Results** **n (%)**
**Gender**	
Male	97 (46.9)
Female	110 (53.1)
**Race**	
Malay	83 (40.1)
Chinese	102 (49.3)
Indian	22 (10.6)
**Education Level**	
Never been to school	3 (1.4)
Primary school	47 (22.7)
Secondary school	113 (54.6)
Tertiary education	44 (21.3)
**Chronic medical illness**	
Diabetes mellitus	130 (62.8)
Hypertension	167 (80.7)
Dyslipidaemia	145 (70.0)
Stroke	11 (5.3)
Heart disease	21 (10.1)
Gout	11 (5.3)
Arthritis	10 (4.8)
Obesity	4 (1.9)
**Knowing someone with dementia**	
Yes	31 (15.0)
No	167 (80.7)
Not sure	9 (4.3)
**Caring for someone with dementia**	
Yes	17 (8.2)
No	190 (91.8)
**Family members diagnosed with dementia**	
Yes	11 (5.3)
No	196 (94.7)

### Risk of developing MCI among the study participants

Based on the total score of the TUA-WELLNESS questionnaire, 39.1% had a high risk of developing MCI, while the remaining 60.1% had a low risk.

### Awareness level of dementia among study participants

The majority (92.8%) of the participants had a low level of dementia awareness (refer to Table 2).

**Table 2:** Awareness level of dementia among the participants (*N* = 207)

**Awareness Level**	**Results** **n (%)**
Low (score 0 to 5)	192 (92.8%)
Medium (score 5)	11 (5.3%)
High (score ≥ 6)	4 (1.9%)

**Fig. 2:**
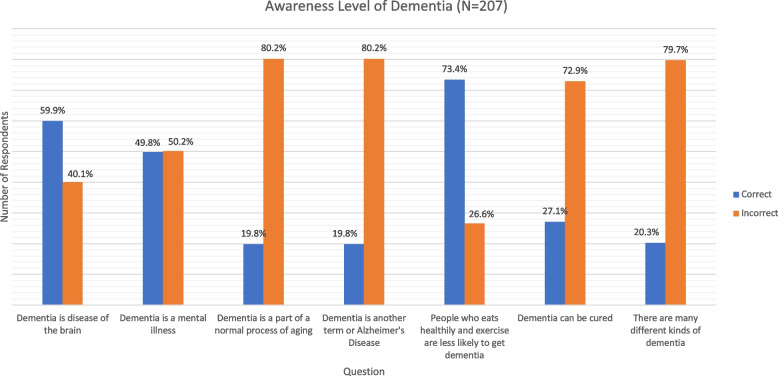
Individual items on awareness level of dementia (*N* = 207)

On individual items (Fig. 2), only 19.8% of participants were able to correctly answer the critical statement (*dementia is a part of the normal process of aging*). Among 207 participants, slightly more than half (59.9%) incorrectly thought that dementia is a disease of the brain and almost half (49.8%) correctly stated that dementia is not a mental illness (refer Fig. 2).

### Consultation related to dementia risk with the healthcare provider

Only 4.8% of the patients had some discussion about the risk of cognitive impairment with their physicians, while 8.2% were unsure. The majority of the participants (93.2%) did not initiate or share their concerns regarding dementia with their physicians, with 2.4% unsure if they had ever discussed this topic before.

### Association of total awareness score among clinical and demographic factors

There were statistically significant differences in scores on dementia awareness between the age groups, risk of cognitive impairment and presence of cardiovascular disease. The awareness score was higher in the younger age group compared to the middle and older age groups (*p* = 0.006).

Those with reported past cardiovascular events (i.e. stroke and ischaemic heart disease) and with high risk of MCI have lower awareness scores with *p* = 0.045 and < 0.01, respectively.

However, gender, race, level of education, all chronic illnesses on their own and cardiovascular risk factor showed no significant differences in scores for awareness of dementia, as summarised in Table 3.

**Table 3:** Differences of total awareness score according to clinical and demographic factors

**Demographic data**	**Total awareness score**	***P*-value**
1. Age group		
• Youngest Old (61–74 years old)	3.00 (IQR 2,4)	0.006
• Middle and Oldest Old (≥ 75 years old)	2.00 (IQR 0.5,4)	
2. Gender		
• Male	3.00 (IQR 2,4)	0.408
• Female	3.00 (IQR 2,4)	
3. Race		
• Malay	3.00 (IQR 2,4)	0.250
• Non-Malay	3.00 (IQR 3,4)	
4. Education level		
• Never been to school	3.00 (IQR 2,4)	0.755
• Primary school and above	3.00 (IQR 2,4)	
5. Chronic Illness		
I. Diabetes Mellitus (DM)		
• Present	3.00 (IQR 1.75,4)	0.139
• Not present	3.00 (IQR 2,4)	
II. Hypertension (HTN)		
• Present	3.00 (IQR 2,4)	0.083
• Not Present	3.00 (IQR 2,4)	
III. Dyslipidaemia		
• Present	3.00 (IQR 2,4)	0.162
• Not Present	3.00 (IQR 2,4)	
IV. Stroke		
• Present	3.00 (IQR 1,3)	0.569
• Not present	3.00 (IQR 2,4)	
V. Heart disease		
• Present	2.00 (IQR 1,3)	0.088
• Not present	3.00 (IQR 2,4)	
VI. Arthritis		
• Present	3.00 (IQR 2,4)	0.709
• Not present	3.00 (IQR 2,4)	
VII. Obesity		
• Present	3.00 (IQR 2,3.5)	0.874
• Not Present	3.00 (IQR 2,4)	
VIII. Cardiovascular Disease (Stroke and Heart Disease)		
• Present	3.00 (IQR 1,3)	0.045
• Not Present	3.00 (IQR 2,4)	
XI. Cardiovascular Risk Factors (DM, HTN and Dyslipidaemias)		
• Present	3.00 (IQR 2,4)	0.301
• Not Present	5.00 (IQR 3,5)	
6. Risk of cognitive impairment		
• High risk	3.00 (IQR 2,4)	0.001
• Low risk	2.00(IQR 1,3)	

Significant at *p* < 0.05

*IQR* Interquartile range

## Discussion

To the best of our knowledge, our study is the first to assess awareness of dementia among patients who are at risk, i.e. patients with multiple non-communicable diseases attending primary care. As the nation ages, and given the high local NCD burden which receives treatment at public primary care facilities, there is a clear need to evaluate the magnitude of the potential effects of dementia. The elderly population in Malaysia in 2021 is reported at 11.2% of the total population. Despite this, only 15% of our study respondents reported exposure to people with dementia, and this is much lower when compared to surveys in western countries, 45–61% in Northern Ireland and Canada, respectively [8, 22].

All respondents who participated in our study had been diagnosed with at least one chronic condition (diabetes mellitus, hypertension or dyslipidaemia). These three chronic diseases are common in this population alongside other lifestyle-related conditions, such as obesity, which share the same risk factors and pathophysiology as cardiovascular diseases [23, 24]. Malaysia has the highest prevalence of obesity in South East Asia, where half of its population are overweight, and nearly 20% are in the obese category [25].

Close to 40% of our study population had a high risk of developing mild cognitive impairment (MCI). Earlier, a hospital-based study by Razali et al. reported a high prevalence of MCI among elderly patients attending specialist outpatient clinics (i.e. 64.7% in 2012 and 55.6% in 2014) [26]. Similarly, our findings correspond to a study in Saudi Arabia where MCI among elderly patients recorded a prevalence of 38.6% [27].

Regarding awareness, our study population demonstrated that more than 90% of the respondents had an overall limited basic knowledge of dementia. The prevalence is double of a survey in Northern Ireland in 2012, which reported that 49% of the elderly study population had a low level of awareness of dementia [8]. This trend was also seen in Australia and most surveys worldwide [28]. Conversely, only 15.8% of the elderly Singaporeans have poor dementia awareness [29]. However, the different results in prevalence could be due to the different non-standardised assessment tools used in these studies.

The majority of the respondents (80%) incorrectly viewed dementia as a part of the normal aging process. We took this as a critical question that needed a correct answer as to acknowledge dementia as a pathological disease and not associated with age-related memory decline [15]. Most studies showed a high prevalence of respondents still viewing dementia as a normal part of aging, with findings ranging from 40–70% (Australia, Northern Ireland, Shanghai, China, Canada) [8, 18, 22, 30]. We postulate that the lower numbers were probably due to the study population age groups recruited, whilst our study focused specifically among the elderly aged 6o and above.

Our findings were similar to studies from China and South Korea, where respondents from younger age groups showed better knowledge and awareness compared to the older age respondents [30, 31]. However, Tan et al., in their analysis, concluded that age differences did not influence dementia awareness levels and suggested that this was probably attributable to the analysing process of the respondents [29].

Our study demonstrated that the total awareness scores were not associated with respondents’ education levels. Conversely, most studies found that the level of education was a factor in increasing the level of awareness [8, 30, 31]. We postulate that better socioeconomic status and access to information influenced awareness levels [31]. We also could not elicit any association between the level of awareness with gender and ethnic background. These findings were similar to a study conducted among a diverse group of people in America [32].

Our study focused on patients with a higher risk of developing dementia. Prevention strategies for dementia mainly focus on controlling modifiable risk factors such as hypertension, diabetes, dyslipidaemia and obesity, which are mainly cardiovascular risks. For these reasons, those with chronic diseases should be more aware of their risk of developing dementia. However, we could not elicit any significant association between these chronic conditions and the level of awareness (*p* > 0.05). In contrast, the study showed lower awareness scores among respondents with established cardiovascular complications, i.e., stroke and ischaemic heart disease (*p* < 0.05). Even more worrying, our study showed that elderly with a high risk of MCI, a precursor of dementia, had significantly lower dementia awareness scores (*p* < 0.05).

In their study conducted in Australia, Smith and colleagues revealed that people were unaware of the link between cardiovascular disease management as a way to prevent dementia [33]. In contrast, researchers in China concluded that most respondents correctly identified the link between lifestyle factors such as alcohol use, smoking, and leisure activities related to dementia. However, the role of chronic medical conditions that may cause dementia is often underestimated [30]. This could be partly because public health campaigns on dementia did not emphasise chronic diseases as a direct cause of dementia [34].

More evidence is emerging to advocate risk factor management in reducing the incidence of dementia. Previously, single-domain dementia and cognitive impairment prevention trials have yielded mainly weak outcomes [35]. Cognitive impairment and dementia are multifactorial disorders and require multidomain intervention focusing on several risk factors and disease mechanisms simultaneously for optimum preventive outcomes. Successful cardiovascular disease and type 2 diabetes preventive trials have highlighted the necessity of a multidomain approach [36]. The randomised controlled trial conducted by FINGER-Lancet 2015 suggested that multidomain intervention, which ﻿consisted of nutritional guidance, exercise, cognitive training, and intensive monitoring of vascular and metabolic risk factors, could improve or preserve cognitive functioning in at-risk older people from the general population [37].

Regular medical consultations help in the early detection of dementia [38, 39]. The lack of dementia awareness among health care providers may also contribute to an overall low level of awareness of dementia [39]. A study done in Virginia, United States, reported that most doctors only educate their patients on dementia after diagnosis rather than explaining to them when they are at risk [40]. The majority of the respondents in this study who regularly visited their general practitioner (GP) for chronic disease management had never requested or participated in any discussion with their GP regarding dementia or MCI. As the proportion of elderly Malaysians continues to increase, the assessment of the preparedness of the healthcare service to cope is crucial. Apart from improving dementia knowledge among primary care providers, future healthcare providers, i.e. medical students must be prepared to cope with the challenge of caring for the aging Malaysian population [41]. Early detection to enable pre-emptive management of dementia among at-risk populations is critical.

### Limitations

One of the limitations of this study is that potential participants were recruited via a telephone call. The participants had the option to not answer the telephone call at the point of recruitment. Opdenakker and colleagues cited that one possible reason could be the distraction by the environmental activities, rendering the interviewer at a disadvantage in creating a pleasant and conducive interview environment [42]. Other possible reasons would be refusing to talk to strangers, language barriers and long interview sessions. To overcome this issue, we sent an e-poster to each participant 2 days before conducting the telephone interview. This was done to introduce them to the researchers and the study purpose and allow the participants to prepare themselves for the interview session. Then, every time before starting the interview session, we informed the respondents that the interview would only require around 5 to 10 min to reduce the possibility of them rejecting or hanging up the phone.

Another limitation is that all clinical variables were self-reported by the respondents and not objectively measured. This may contribute to self-report response bias. Response bias, which stems from the respondent, has an impact on measurement quality by bringing error or a lack of precision into the study [43]. For various reasons, respondents may be unwilling or unable to provide accurate responses [43]. Future studies should include verification of medical conditions by accessing medical records.

Last but not least, participants’ responses may have been influenced by searching for information and answers regarding dementia on the internet since we had sent them a poster of our study prior to contacting them, and may contribute to bias.

### Recommendations

Based on this study, people with chronic diseases at higher risk for MCI had limited knowledge of dementia. Efforts to increase awareness of the role of lifestyle factors in dementia risk reduction need to be urgently addressed, focusing on educating the public about the role of modifiable risk and preventive factors. Primary care physicians should address the elevated risk of MCI and subsequent dementia with their patients receiving chronic care to be better aware of the condition and leverage this information to increase their motivation to reduce their risk of developing dementia.

Future studies in multicentre trials should be conducted with patients and primary care doctors representing urban and rural communities to assess awareness levels and perhaps recommend targeted suggestions for improving awareness in these populations.

## Conclusion

Awareness of dementia is low among elderly patients with a potentially high risk of developing MCI. Efforts to improve awareness on dementia should focus on primary care doctors engaging with at-risk elderly patients to initiate discussion regarding dementia risk while managing modifiable risk factors, i.e. hypertension control, diabetes, dyslipidaemia and obesity.

## Data Availability

The datasets generated during and analysed during the current study are not publicly available due to institutional research policy, but are available from the corresponding author on reasonable request.

## Abbreviations

HCTM: Hospital Canselor Tuanku Mukhriz
TUA: Towards Useful Aging
MCI: Mild Cognitive Impairment
WHO: World Health Organization
PLWD: People living with dementia
SEA: South East Asia
FBS: Fasting blood sugar
SD: Standard deviation
GP: General practitioner
KPPC: Klinik Primer PPUKM Cheras
UKM: Universiti Kebangsaan Malaysia
